# Length of stay of post-acute care: determinants and differences between traditional medicare and medicare advantage

**DOI:** 10.1093/haschl/qxaf195

**Published:** 2025-10-13

**Authors:** Dian Luo, Ying (Jessica) Cao, Mariétou H Ouayogodé, Wan-chin Kuo, John Mullahy, Marguerite E Burns

**Affiliations:** Wisconsin Center for Education Research, University of Wisconsin-Madison, Madison, WI 53706, United States; Department of Population Health Sciences, University of Wisconsin-Madison, Madison, WI 53726, United States; Center for Demography and Health of Aging, University of Wisconsin-Madison, Madison, WI 53726, United States; Health Innovation Program, University of Wisconsin-Madison, Madison, WI 53726, United States; Department of Population Health Sciences, University of Wisconsin-Madison, Madison, WI 53726, United States; Center for Demography and Health of Aging, University of Wisconsin-Madison, Madison, WI 53726, United States; School of Nursing, Madison, WI 53705, United States; Department of Population Health Sciences, University of Wisconsin-Madison, Madison, WI 53726, United States; Center for Demography and Health of Aging, University of Wisconsin-Madison, Madison, WI 53726, United States; Department of Population Health Sciences, University of Wisconsin-Madison, Madison, WI 53726, United States; Center for Demography and Health of Aging, University of Wisconsin-Madison, Madison, WI 53726, United States

**Keywords:** traditional Medicare, Medicare Advantage, inpatient rehabilitation facilities, post-acute care, health care equity

## Introduction

Post-acute care (PAC) in inpatient rehabilitation facilities (IRFs) is a critical component of recovery for older adults. With Medicare Advantage (MA) enrollment surpassing half of eligible beneficiaries currently, understanding whether length of stay (LOS) is comparable between traditional Medicare (TM) and MA is central to health care parity in PAC utilization. We define health care parity as similar utilization for patients with similar clinical need.^1^ Substantial variation in Medicare use and spending persists, raising concerns about consistent access and practice patterns.^2-6^ However, most IRF studies emphasize costs and payment effects rather than whether patient, facility, or regional factors explain LOS differences between TM and MA.^7,8^ We address this gap by quantifying the relative contributions of clinical, demographic, facility, and regional factors to LOS for common IRF conditions. We also assess whether TM's prospective payment system (PPS), which anchors LOS to clinical conditions, exerts a spillover effect on MA.

## Methods

We analyzed 2019-2020 Inpatient Rehabilitation Facility–Patient Assessment Instrument (IRF-PAI) data from the Uniform Data System for Medical Rehabilitation. Episodes were categorized by discharge month into pre-pandemic (January 2019–March 2020) and pandemic (April–December 2020) periods. The outcome was LOS, modeled as a count variable using Poisson regression. Covariates included patient, facility, and regional factors. To assess the relative importance of explanatory factors, we applied dominance analysis (DA), which quantifies each factor's contribution to overall model fit. DA allows comparison of patient clinical conditions, demographics, facility attributes, and regional factors in explaining LOS variation. We also evaluated whether regional characteristics, such as regional MA penetration and Herfindahl-Hirschman Index (HHI), captured the same variation as regional fixed effects. We focused on 4 common conditions among older adults: stroke (*n* = 127 685), hip fracture (*n* = 64 241), cardiac (*n* = 33 116), and joint replacement (*n* = 17 913). The final analytic sample included 242 955 patient episodes after excluding cases with non-Medicare coverage, age <65, non-initial admissions, non-home pre-hospital settings, program interruptions, outlier LOS (<3 or >30 days), and in-hospital deaths. Further details are provided in the Supplement.

## Results

Across all conditions and Medicare types, clinical factors were the dominant determinants of LOS, explaining roughly 90% or more of variation (Table 1). For stroke patients, clinical conditions accounted for nearly 98% of LOS for both TM and MA. Similar patterns were observed for hip fracture, cardiac, and joint replacement episodes, though the relative influence of clinical factors was somewhat lower for joint replacement. Demographics were generally the second most important contributors to LOS, while facility factors played a smaller role. For example, in hip fracture and cardiac episodes, demographics explained 3%-7% of LOS variation, compared with <2% for facility characteristics. Regional factors, captured by regional fixed effects, explained more variation than explicit regional measures such as regional MA penetration and HHI. For stroke, regional fixed effects accounted for about 0.5%-0.6% of LOS variation, vs <0.2% for regional characteristics. This pattern held across conditions, underscoring that unobserved local factors mattered more than market competition metrics. Together, these results suggest that PPS regulation in TM tightly anchors LOS, with spillover to MA beneficiaries. Detailed regression and DA results are provided in the Supplement.

**Table 1.** Dominance analysis of LOS with regional fixed effects.

	Stroke			Hip fracture			Cardiac			Joint replacement		
	Dominance statistics	Standardized dominance statistics	Ranking	Dominance statistics	Standardized dominance statistics	Ranking	Dominance statistics	Standardized dominance statistics	Ranking	Dominance statistics	Standardized dominance statistics	Ranking
**Model with regional fixed effects**												
**Overall**												
MA status and pandemic period	0.0002	0.0015	5	0.0001	0.0023	6	0.0003	0.0051	5	0.0006	0.0088	5
Clinical condition	0.1291	0.9833	1	0.0505	0.9030	1	0.0540	0.9422	1	0.0576	0.9012	1
Demographics	0.0005	0.0037	4	0.0039	0.0696	2	0.0017	0.0304	2	0.0034	0.0538	2
Facility factors	0.0007	0.0053	2	0.0005	0.0096	4	0.0005	0.0084	4	0.0007	0.0103	4
Regional fixed effects	0.0006	0.0047	3	0.0007	0.0127	3	0.0006	0.0096	3	0.0014	0.0213	3
Discharge month	0.0002	0.0014	6	0.0002	0.0027	5	0.0002	0.0043	6	0.0003	0.0045	6
**TM**												
Pandemic period	0.0002	0.0012	6	0.0001	0.0022	6	0.0002	0.0032	6	0.0006	0.0090	4
Clinical condition	0.1323	0.9833	1	0.0502	0.9016	1	0.0526	0.9408	1	0.0572	0.9147	1
Demographics	0.0005	0.0037	4	0.0039	0.0705	2	0.0018	0.0330	2	0.0030	0.0478	2
Facility factors	0.0007	0.0049	3	0.0004	0.0076	4	0.0004	0.0075	4	0.0002	0.0027	6
Regional fixed effects	0.0007	0.0054	2	0.0009	0.0154	3	0.0007	0.0117	3	0.0013	0.0205	3
Discharge month	0.0002	0.0015	5	0.0001	0.0027	5	0.0002	0.0039	5	0.0003	0.0054	5
**MA**												
Pandemic period	0.0002	0.0012	6	0.0001	0.0020	6	0.0001	0.0022	6	0.0004	0.0056	6
Clinical condition	0.1228	0.9802	1	0.0526	0.8939	1	0.0615	0.9512	1	0.0607	0.7776	1
Demographics	0.0007	0.0056	3	0.0041	0.0694	2	0.0017	0.0256	2	0.0074	0.0954	2
Facility factors	0.0006	0.0051	4	0.0007	0.0125	4	0.0001	0.0022	5	0.0034	0.0438	4
Regional fixed effects	0.0008	0.0061	2	0.0008	0.0141	3	0.0004	0.0064	4	0.0049	0.0632	3
Discharge month	0.0002	0.0018	5	0.0005	0.0080	5	0.0008	0.0124	3	0.0011	0.0145	5
**Model with regional characteristics**												
**Overall**												
MA status and pandemic period	0.0002	0.0013	6	0.0001	0.0020	6	0.0002	0.0040	6	0.0006	0.0088	4
Clinical condition	0.1293	0.9864	1	0.0508	0.9114	1	0.0541	0.9478	1	0.0580	0.9172	1
Demographics	0.0005	0.0037	3	0.0039	0.0702	2	0.0017	0.0307	2	0.0035	0.0546	2
Facility factors	0.0007	0.0053	2	0.0005	0.0097	3	0.0005	0.0081	3	0.0006	0.0096	3
Regional characteristics	0.0002	0.0018	4	0.0002	0.0040	4	0.0003	0.0050	4	0.0003	0.0055	5
Discharge month	0.0002	0.0014	5	0.0002	0.0027	5	0.0002	0.0044	5	0.0003	0.0045	6
**TM**												
Pandemic period	0.0001	0.0011	6	0.0001	0.0020	6	0.0002	0.0027	6	0.0005	0.0085	3
Clinical condition	0.1325	0.9869	1	0.0504	0.9109	1	0.0527	0.9482	1	0.0575	0.9310	1
Demographics	0.0005	0.0037	3	0.0040	0.0715	2	0.0018	0.0332	2	0.0030	0.0490	2
Facility factors	0.0007	0.0052	2	0.0005	0.0088	3	0.0004	0.0079	3	0.0002	0.0039	5
Regional characteristics	0.0002	0.0016	4	0.0002	0.0041	4	0.0002	0.0040	4	0.0001	0.0021	6
Discharge month	0.0002	0.0015	5	0.0001	0.0027	5	0.0002	0.0039	5	0.0003	0.0055	4
**MA**												
Pandemic period	0.0001	0.0010	6	0.0001	0.0017	6	0.0001	0.0020	6	0.0006	0.0083	6
Clinical condition	0.1231	0.9839	1	0.0528	0.9019	1	0.0617	0.9551	1	0.0619	0.8058	1
Demographics	0.0007	0.0059	2	0.0042	0.0711	2	0.0016	0.0254	2	0.0080	0.1039	2
Facility factors	0.0007	0.0054	3	0.0008	0.0140	3	0.0002	0.0025	5	0.0036	0.0472	3
Regional characteristics	0.0002	0.0019	4	0.0002	0.0033	5	0.0002	0.0027	4	0.0016	0.0209	4
Discharge month	0.0002	0.0018	5	0.0005	0.0081	4	0.0008	0.0123	3	0.0011	0.0139	5

## Discussion

This study examined the relative importance of clinical, demographic, facility, and regional factors in explaining LOS in IRFs, with a focus on whether these drivers differ between TM and MA. Clinical conditions overwhelmingly explained LOS in both programs, accounting for roughly 90% or more of variation across conditions. For stroke, clinical factors explained nearly 98% of LOS, demonstrating that PPS adjustments effectively anchor LOS to patient need. Beyond clinical conditions, however, some differences emerged. Demographics generally ranked as the second most important determinants of LOS, surpassing facility characteristics. Their influence varied somewhat by condition and program. Regional fixed effects explained more variation than explicit regional characteristics such as regional MA penetration or HHI. This indicates that unobserved local practice patterns are more influential than competition-based measures in shaping LOS. These patterns suggest that while PPS regulation in TM strongly anchors LOS, secondary drivers of variation are not identical across TM and MA. Together, these findings highlight that PPS contributes to consistency in LOS across programs, but demographic and regional factors shape variation differently between TM and MA. Monitoring how these non-clinical factors affect LOS in each program is essential for ensuring fair and efficient PAC delivery.

## Contribution statement

Study concept and design: Y.J.C. and D.L. Acquisition of data: Y.J.C. Analysis and interpretation of data: all authors. Drafting of the manuscript: D.L. Critical revision of the manuscript for important intellectual content: all authors.

## Supplementary Material

qxaf195_Supplementary_Data
